# The Function of Transforming Growth Factor 2 in Facilitating Inflammasome Activation to Enhance the Development of Myopia via Complement System

**DOI:** 10.3390/cells14161295

**Published:** 2025-08-20

**Authors:** Sheng-Chun Lin, Yu-An Hsu, Chi-Fong Lin, Chih-Sheng Chen, Peng-Tai Tien, Yao-Chien Wang, Ching-Yao Chang, En-Shyh Lin, Jamie Jiin-Yi Chen, Ming-Yen Wu, Hui-Ju Lin, Lei Wan

**Affiliations:** 1 Graduate Institute of Biomedical Sciences, China Medical University, Taichung 406040, Taiwan; u106023003@cmu.edu.tw; 2 School of Chinese Medicine, China Medical University, Taichung 406040, Taiwan; annhsu007@gmail.com; 3 Department of Chemistry, National Central University, Taoyuan 320317, Taiwan; 4 Ph.D. Program for Health Science and Industry, China Medical University, Taichung 406040, Taiwan; j331475@gmail.com; 5 Department of Food Nutrition and Health Biotechnology, Asia University, Taichung 413305, Taiwan; pluto915@mail2000.com.tw; 6 Division of Chinese Medicine, Asia University Hospital, Taichung 413505, Taiwan; 7 School of Medicine, China Medical University, Taichung 406040, Taiwan; miketien913@gmail.com; 8 Eye Center, China Medical University Hospital, Taichung 404327, Taiwan; mnxxvy19@gmail.com (J.J.-Y.C.); stevenwu9@hotmail.com (M.-Y.W.); 9 Department of Emergency Medicine, Taichung Tzu Chi Hospital, Taichung 42743, Taiwan; gbjjeng@gmail.com; 10 Department of Medical Laboratory Science and Biotechnology, Asia University, Taichung 41354, Taiwan; cychang@asia.edu.tw; 11 Department of Beauty Science, National Taichung University of Science and Technology, Taichung 403027, Taiwan; eslin@nutc.edu.tw; 12 Department of Obstetrics and Gynecology, China Medical University Hospital, Taichung 40402, Taiwan

**Keywords:** myopia, TGF-β2, complement system, CD55, inflammasome, NF-κB, gene therapy

## Abstract

Myopia is one of the major public health conditions with significant complications. This study investigates the role of transforming growth factor (TGF)-β2, complement activation, and inflammasome pathways in myopia progression using a Brown Norway rat model. Myopia was induced, and complement regulation was manipulated using gene therapy via adeno-associated virus (AAV) vectors delivering CD55 or CD55 siRNA. Results showed that TGF-β2 exacerbated myopia by upregulating complement components C3 and C5, suppressing CD55, and activating inflammasome pathways through nuclear factor (NF)-κB signaling, leading to axial elongation and increased refractive errors. Overexpression of CD55 via AAV gene therapy effectively counteracted these effects, reducing axial length elongation and inflammation by suppressing inflammasome markers interleukin (IL)-1β and NLR family pyrin domain containing 3 (NLRP3), as confirmed by real-time quantitative PCR and immunofluorescence analyses. Conversely, silencing CD55 intensified TGF-β2-induced effects, further promoting axial elongation and inflammation. These findings highlight the critical role of CD55 in modulating TGF-β2-driven complement and inflammasome activation during myopia progression. The study suggests that gene therapy targeting CD55 could serve as a novel therapeutic strategy to mitigate myopia and related inflammatory processes, offering a promising avenue for managing this significant public health challenge.

## 1. Introduction

Refractive error significantly contributes to visual impairment. Myopia is the common cause of considerable pressure on healthcare systems and economies worldwide [1]. Approximately 153 million individuals over the age of 5 experience varying degrees of distant vision impairment; of these, eight million individuals are blind as a result of untreated refractive errors [2]. Myopia is a prevalent yet insufficiently managed ocular condition. While glasses, contact lenses, and refractive surgery can effectively correct most cases of myopia, roughly 33% of visual impairment cases are still caused by uncorrected refractive errors [3]. High myopia is a key factor that leads to serious cataracts, macular choroidal degeneration, retinal detachment, and glaucoma. Patients with myopia of −6 D or less have a 3.2% annual risk of retinal detachment. The incidence of macular choroidal neovascularization in this group is 9-fold greater [4,5]. Myopia has been observed in the global initiative aimed at eliminating preventable blindness [6].

Transforming growth factor β (TGF-β) is a multifunctional cytokine with essential function in modulating extracellular matrix synthesis, cellular development, inflammation, and apoptosis [7]. TGF-β serves a crucial role in controlling the synthesis of matrix metalloproteinases, collagen, and proteoglycan in the extracellular matrix [8]. A previous study demonstrated the presence of TGF-β isoforms within the mammalian sclera [9]. Another study has connected heightened TGF-β2 function in the sclera to the progression of myopia generated by the use of negative lenses in guinea pigs [10]. Clinical investigations indicate that patients with high myopia have elevated amounts of TGF-βs in the vitreous humor [11]. Animal experiments have demonstrated that an increase in TGF-βs inside the choroid is associated with the elongation of the eye [12]. TGF-β2 increases matrix metalloproteinase 2 (MMP2) and decreases collagen I expression, promoting myopia [13]. TGF-β1 regulates the expression of MMP2 in fibroblasts by activating nuclear factor κB (NF-κB) [14], and MMP2 has the ability to break down collagen types I, III, and V, leading to the restructuring of the sclera [14].

The initiation of the complement system within the human organism is carefully managed to minimize overstimulation and the associated negative effects of inflammation. It is maintained at a steady and low turnover rate and plays an important function in preserving ocular immune privilege. Various eye illnesses, including age-related macular degeneration, autoimmune uveitis, diabetic retinopathy, and glaucoma, exhibit abnormal regulation of the complement system [15]. The deregulation of the complement system has been associated with the development of myopia. Patients with pathologic myopia (−8 D to −25 D) showed significantly higher levels of C3 (*p* = 0.004) and CH50 (*p* < 0.001) [16]. Myopic guinea pigs who received defocused negative lenses had considerably increased amounts of C1q, C3, and C5b-9 in their sclera [17]. Activation of the complement system may promote extracellular matrix remodeling and contribute to myopia development [17]. A meta-analysis was performed on eight different transcriptome databases to evaluate the link between lens-induced or form-deprivation myopia in chicks. The study found that the activation of the complement system is one of the biochemical pathways implicated in the advancement of myopia [18]. It is determined that the eyes are highly susceptible to disruption of the complement system [18]. Hence, abnormal activation of the complement system significantly contributes to the development of myopia. CD55 is a glycoprotein that is attached to the cell membrane by glycosylphosphatidylinositol. CD55 inhibits the activation of C3 and C5 by preventing the synthesis of C3 and C5 convertases and allowing their degradation. CD55 inhibits the activation of the complement system [19,20,21].

Inflammasomes serve a crucial role in innate immunity by stimulating the development and synthesis of cytokines that promote inflammation, IL-1β and IL-18 [22]. Studies highlight a significant crosstalk between the complement system and inflammasome activation [23,24]. For instance, complement activation products like C3a and C5a can act as signaling molecules, enhancing inflammasome activation [23,24]. C5a has been shown to bind to its receptor, C5aR, promoting NLRP3 inflammasome assembly and activation [23,24]. Inflammasome-derived cytokines can influence complement activation by activating NF-κB [25,26], creating a feedback loop that amplifies the immune response.

Based on the provided information, TGF-β, the complement system, and the inflammasome all involve molecules that possess the ability to facilitate the development of myopia. Our intention is to assess the possible correlation between these three factors in the development of myopia. By elucidating these pathways, we can identify potential therapeutic targets to modulate scleral remodeling and inflammation, potentially leading to new treatments for myopia.

## 2. Materials and Methods

### 2.1. Vector Production, Purification and Injection

CD55 AAV vector (Rat) (CMV), AAV Blank control vector (CMV), CD55 AAV siRNA pooled vector (Rat), and scrambled AAV siRNA control plasmids were obtained from Applied Biological Materials Inc. (Richmond, BC, Canada). An AAV-DJ helper free packaging system (Cell Biolabs, San Diego, CA, USA) was utilized to generate AAV virus in AAV-100 cells (Cell Biolabs, San Diego, CA, USA). AAV viruses were recovered using ViralBing^TM^ AAV purification kit (Cell Biolabs, San Diego, CA, USA) and titrated by a QuickTiter^TM^ AAV quantitation kit (Cell Biolabs, San Diego, CA, USA).

The rats were administered Zoletil at a dose of 5 mg/0.1 kg body weight for anesthesia and were then positioned on a heating pad to regulate their body temperature. A small opening was created in the sclera near the limbus using a 30-gauge disposable needle. Then, a 33-gauge blunt-tip needle connected to a Hamilton syringe was introduced through the scleral aperture into the vitreous area to provide intravitreal injections. The needle was left in the vitreous cavity for approximately 2–3 s to guarantee a complete injection, following which it was removed. Each eye was administered 1 µL of vector with a titer of 1 × 10^12^ GC/mL.

### 2.2. Myopia Animal Models

Three-week-old male Brown Norway rats were acquired from the National Laboratory Animal Center in Taipei, Taiwan, and randomly allocated to experiment groups; 210 rats were utilized in this investigation and were housed on a 12 h light/12 h dark cycle. Ten rats were assigned to each group for every experiment. All procedures were approved by the Institutional Animal Care and Use Committee of China Medical University (CMUIACUC-2019-173 approved on 29 December 2018). All operations followed the rules for the use of animals in ophthalmic and vision research.

#### 2.2.1. Form-Deprivation Myopia Animal Model

In the monocular form-derived myopia study, fusion of the right eyelid caused myopia to develop. The concentration of TGF-β2 was measured by enucleating the eyes on days 0, 7, 14, and 21 and collecting the vitreous humor, retina, and sclera.

#### 2.2.2. TGF-β-Induced Myopia Animal Model

PeproTech Inc., Cranbury, NJ, USA, provided recombinant TGF-β1 (100-21), 2 (100-35B), and 3 (100-36E), which were dissolved in balanced salt solution (BSS). The diluted solutions were kept at −20 °C until use. The recombinant TGF-βs were injected subconjunctivally on the first, seventh, and fourteenth days. The rats were administered Zoletil (5 mg/0.1 kg) to induce anesthesia. The eye’s surface was sterilized using iodophor and subsequently rinsed extensively with sterile BSS. In the myopic eyes, a total of 1.5 ng of recombinant TGF-βs was injected, while the control eyes were injected with BSS.

The axial lengths (AL) and refractive errors (RE) of the eyes were measured on days 0 and 21 of each study.

### 2.3. Ocular Biometry Assessment

A portable streak retinoscope was used to examine each eye’s refractive status. Tropicamide was administered to cause pupil dilatation. The animals were given Zoletil (5 mg/0.1 kg) to induce anesthesia. Ocular refraction was measured before and after the experiment. The initial experiment used five different degrees of lens. During the second cycle, the refractive condition was measured within a ±0.5-degree range using pupil luminescence measurement. The PacScan 300 Plus instrument from New Hyde Park, NY, USA, was employed to measure the axial lengths using A-scan ultrasonography. A mean value was determined from ten different measurements. After the study, the animals were euthanized via CO_2_ asphyxiation. The eyes were enucleated and either paraffin-embedded for immunofluorescence labeling or protein-extracted for Western blotting.

### 2.4. Determination the Level of Transforming Growth Factor β2 (TGF-β2)

Using RIPA buffer supplemented with a combination of protease and phosphoprotease inhibitors (Roche, West Sussex, UK), vitreous humor, retina, and sclera tissues were homogenized; 50 μg total protein was subjected to treatment with Sample Activation 1 (DY010; R&D systems, Minneapolis, MN, USA). The TGF-β2 concentration in the tissue lysate was determined by a Mouse/Rat/Canine/Porcine TGF-β2 Quantikine ELISA Kit (MB200; R&D systems, Minneapolis, MN, USA).

### 2.5. Immunofluorescence Staining

The eyes were submerged in a solution of 4% paraformaldehyde (Sigma-Aldrich Corp., St. Louis, MO, USA) in PBS overnight before being embedded in paraffin. Tissue blocks were chopped into 3 μm slices and placed on glass slides. The sections were treated to remove paraffin and then prepared for antigen retrieval by incubating them in a buffer solution (Epitope Retrieval Solution, Leica Biosystems, Buffalo Grove, IL, USA) for 20 min. They were subsequently submerged in a 3% hydrogen peroxide solution for 30 min (Polymer Detection System, Leica Biosystems, Buffalo Grove, IL, USA). After that, the sections were blocked with a solution containing 5% normal goat serum in PBS for 30 min at room temperature. Finally, they were incubated overnight at 4 °C with the specific primary antibodies against CD55 (sc-9156, RRID:AB_2075970, Santa Cruz, TX, USA), transforming growth factor β (TGF-β) (ab66043, RRID:AB_1143428, Abcam, Cambridge, UK), C3 (GTX72994, RRID:AB_374787, GeneTex, Irvine, CA, USA), collagen type I (collagen 1) (GTX20292, RRID:AB_384293, GeneTex, Irvine, CA, USA), C5 (GTX33052, GeneTex, Irvine, CA, USA), C5b-9, IL-1β (ab9722, RRID: AB_308765, Abcam), matrix metalloproteinase-2 (MMP-2) (ab37150, RRID:AB_881512, Abcam, Cambridge, UK), TNF-α (BS1857, RRID:AB_1662107, Bioworld, Dublin, OH, USA), NFκB (ab16502, RRID:AB_443394, Abcam) and NLRP3 (13158, RRID:AB_2798134, Cell Signaling Technology, Danvers, MA, USA). The slides were incubated with goat anti-Rabbit IgG (GTX213110-04 RRID:AB_2887579, Genetex) conjugated to biotin (Alexa Fluor 488 or 546) or Cy™3 (11-165-003 RRID:AB_2338000, Jackson ImmunoResearch Inc., West Grove, PA, USA) for 1 h at room temperature the next day. A confocal spectral microscope, equipped with a white light laser, was utilized as imaging equipment to take retinal pictures from the posterior segment of the eyes at magnifications of 20× or 63× (Leica TCS SP8 X).

### 2.6. Cell Culture

The human retinal pigment epithelial cells (ARPE-19) were acquired from the Bioresource Collection and Research Center in HsinChu, Taiwan (BCRC; BCRC-60,383). The cells were grown in Dulbecco’s Modified Eagle Medium (DMEM) supplemented with 10% fetal bovine serum (FBS) at 37 °C and 5% CO_2_. The medium was changed every three to four days. For TGF-β treatment, the cells were distributed evenly in 6-well plates with a density of 1 × 10^5^ cells per well with the medium changed every other day for 21 days. They were then exposed to TGF-β1, 2, or 3 at a concentration of 10 ng/mL, or a combination of TGF-β2 at 10 ng/mL and SB431542 at a concentration of 5 μM (Sigma-Aldrich), for the specified durations. Cell lysates were obtained to perform real-time quantitative PCR (qPCR) to quantitate gene expression.

### 2.7. Real-Time Quantitative PCR (qPCR)

The total RNA was extracted using the Qiagen RNeasy Mini Kit. To generate cDNA, 5 μg of RNA was reverse-transcribed using the Superscript First Strand Synthesis kit (Invitrogen, Carlsbad, CA, USA). For qPCR analysis, we utilized the Universal Probes Library system (Roche). The specific primer pair and probe number utilized are documented in Supplementary Table S1. The transcript levels were normalized to the expression of glyceraldehyde 3-phosphate dehydrogenase (GAPDH) in all of the samples.

### 2.8. Western Blot Analysis

Retina tissues were collected after animals were sacrificed and homogenized using RIPA buffer, which was supplemented with a mixture of protease and phosphoprotease inhibitors (Roche, West Sussex, UK). The cell lysates were also lysed using radioimmunoprecipitation assay (RIPA) buffer. The protein concentrations were measured with the Bradford protein assay (Bio-Rad, Hercules, CA, USA). The extracts (15 μg) were separated by SDS-PAGE and transferred to PVDF membranes (0.45 μm; Millipore, Billerica, MA, USA). The membranes were blocked for an hour in PBST with 5% nonfat milk, and then primary antibodies were added, including p-NFκB (3033, RRID:AB_331284, Cell Signaling Technology, Danvers, MA, USA), Smad2/3 (3102, RRID:AB_10698742, Cell Signaling Technology, Danvers, MA, USA), NFκB (ab16502, RRID:AB_443394, Abcam), p-Smad2(Ser456/467)/Smad3(Ser423/425) (9510, RRID:AB_2193178, Cell Signaling Technology, Danvers, MA, USA), IL-1β (ab9722, RRID: AB_308765, Abcam), NLRP3 (13158, RRID:AB_2798134, Cell Signaling Technology, Danvers, MA, USA), and β-actin (4970, RRID:AB_2223172, Cell Signaling Technology, Danvers, MA, USA), overnight at 4 °C. After three PBST washes, the membranes were treated for one hour at room temperature with anti-mouse or anti-rabbit antibodies coupled with horseradish peroxidase. Proteins of interest were identified using an enhanced chemiluminescence assay kit, following the instructions provided by the manufacturer. Chemiluminescence was used with an ImageQuant LAS4000 mini (GE Healthcare, Little Chalfont, UK) equipment to view the reaction. The NIH in Bethesda, MD, USA, provided ImageJ 1.49 software, which was used to quantify the immunoblots.

### 2.9. Transepithelial Electrical Resistance

We followed the protocol established by Markert et al. [27] with modification. ARPE-19 cells (1 × 10^5^ cells) were seeded onto VTN-N-coated transwell inserts (0.25 μg/mL; ThermoFisher Scientific) placed in 12-well culture plates. The culture medium was replaced every other day for four weeks. Transepithelial electrical resistance (TEER) was measured using an EVOM2 epithelial voltohmmeter (World Precision Instruments, Sarasota, FL, USA). After four weeks, the mean TEER was 72.25 ± 3.32 Ω·cm^2^. Only wells with TEER values around 70 Ω·cm^2^ were used for subsequent experiments.

### 2.10. Statistical Analysis

ANOVA was used to statistically analyze the differences between the experimental groups with the GraphPad Prism program (Version 9, San Diego, CA, USA). Tukey’s multiple comparison tests are suitable to compare treatment groups in pairs. *p* values less than 0.05 were used to determine statistical significance for the means of the data.

## 3. Results

### 3.1. Transforming Growth Factor βs (TGF-βs) in the Development of Myopia

**Figure 1:**
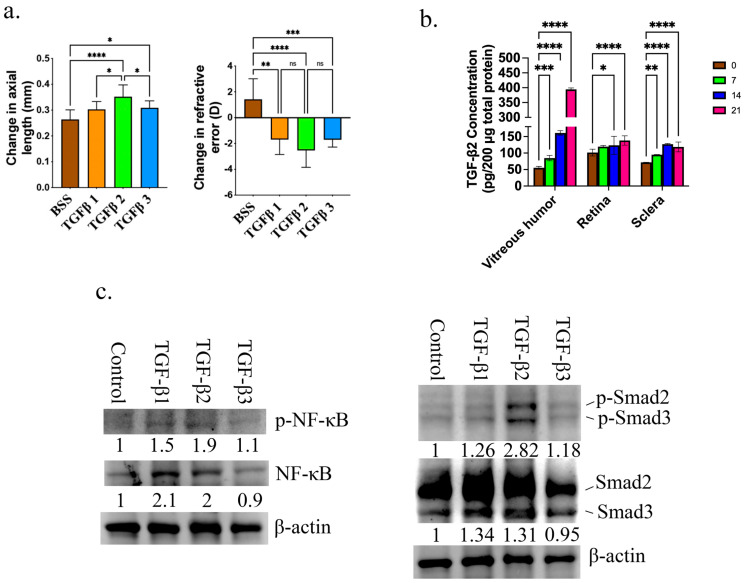
TGF-βs promoted myopia development through activating Smad-2 and NF-κB. (**a**) The changes in axial lengths and refractive errors in eyes treated with balanced salt solution (BSS), TGF-β1, 2 and 3. (**b**) TGF-β2 concentration in vitreous humor, retina and sclera from form-deprivation-induced myopic eyes (day 21). The concentration of TGF-β2 was determined by ELISA. (**c**) Retina tissues were collected on day 21 of TGF-β1-, 2- and 3-treated eyes. Western blot analysis was used to determine the levels of phospho-NFκB (Ser 536), NFκB, phospho-Smad2 (Ser 456/467)/Smad3 (Ser423/425), Smad2/3. β-actin levels were used as an internal benchmark to calculate the relative expression levels using ImageJ software. * *p* < 0.05, ** *p* < 0.01, *** *p* < 0.001, **** *p* < 0.0001. ns: There is no discernible change.

For the study of the impact of TGF-βs on myopia development, 1.5 ng of TGF-β1, TGF-β2, and TGF-β3 was injected subconjunctivally into the right eyes of Brown Norway rats on days 1, 7, and 14. On day 0 and day 21, we assessed the axial length and refractive error. When analyzing the eyes treated with TGF-βs and comparing them to the BSS group, a notable change in axial length and refractive error was observed (Figure 1a). Remarkably, the TGF-β2 group exhibited the most significant axial length elongation and reduction in refractive error (Figure 1a). We also found a significant increase in MMP2 and lower collagen 1 expression in the retina of TGF-β-treated eyes, which further confirmed the development of myopia (Supplementary Figure S1). During the development of myopia, the concentration of TGF-β2 was assessed using a monocular form-deprivation animal model. The vitreous humor, retina, and sclera were collected on days 0, 7, 14, and 21. There was a noticeable rise in the concentration of TGF-β2 over time in all the collected tissue samples, highlighting the significant role of TGF-β2 in the development of myopia (Figure 1b). In addition, there was an observed increase in smad2 and NF-κB activation in the retina treated with TGF-β2, as shown in Figure 1c, compared to TGF-β1 and 3.

**Figure 2:**
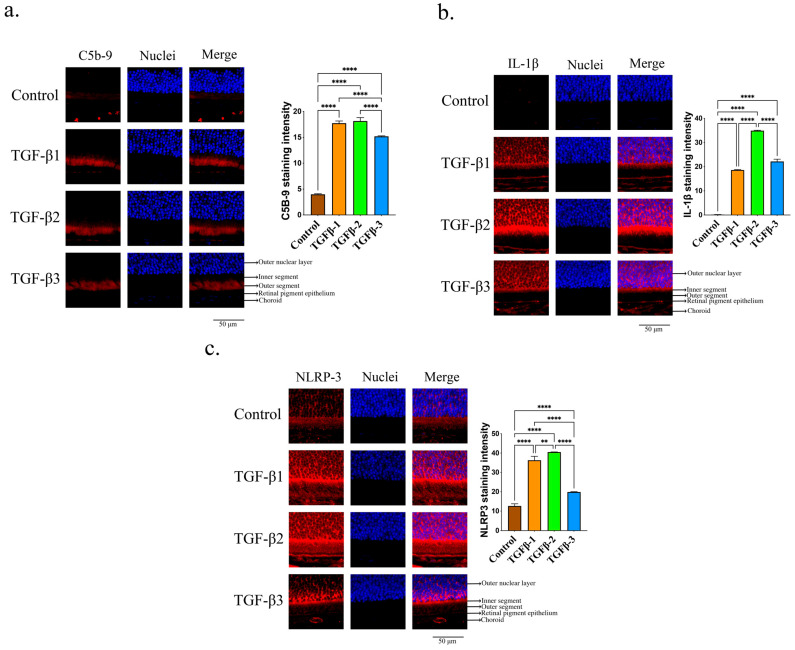
TGF-βs promoted complement activation and inflammasome activation in the eye. Immunofluorescence staining of (**a**) C5b-9, (**b**) IL-1β, and (**c**) NLRP-3 in the retina treated with TGF-β1, 2, or 3. ImageJ software is utilized to ascertain the relative expression levels. In immunofluorescence labeling, ANOVA is utilized to analyze significant differences (*p* < 0.05), whereas Tukey’s multiple comparison tests are employed for paired comparisons between control, TGF-β1-, TGF-β2-, and TGF-β3-treated eyes. ** *p* < 0.01, **** *p* < 0.0001.

Our analysis delved deeper into the activation of the complement system in the eyes treated with TGF-βs. There was a noticeable increase in C5b-9 deposition in the retina of all eyes treated with TGF-βs (Figure 2a). Additionally, there was an observed increase in inflammasome activation in the retina of eyes treated with TGF-βs, as evidenced by the elevated expression of IL-1β (Figure 2b) and NLRP3 (Figure 2c). The levels of IL-1β and NLRP3 in the retina were also confirmed by qPCR (Supplementary Figure S2). It is worth mentioning that TGF-β2 showed the highest level of inflammasome activation when compared to TGF-β1 and 3 (Figure 2b,c). The findings indicated that TGF-β2 facilitated the progression of myopia by augmenting inflammatory responses and activating the complement system.

### 3.2. TGF-β2 Altered the Expression of Complement System Proteins

**Figure 3:**
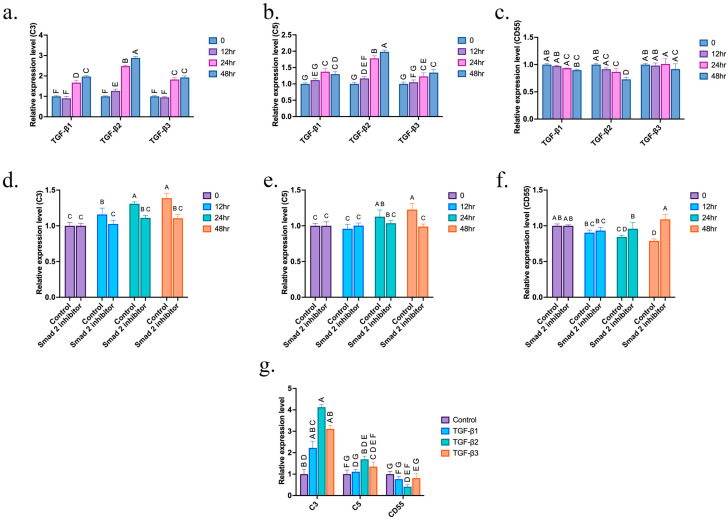
TGF-βs promoted the expression of C3 and C5 while inhibiting the expression of CD55. Retina pigment epithelial cells (ARPE-19) were treated with 10 ng/mL TGF-β1, 2 or 3. The expression levels of (**a**) C3, (**b**) C5 and (**c**) CD55 were determined by qPCR. ARPE-19 cells were treated with 10 ng/mL TGF-β2 or a combination of TGF-β2 at 10 ng/mL and SB431542 at a concentration of 5 μM. The expression levels of (**d**) C3, (**e**) C5 and (**f**) CD55 were determined by qPCR. For comparison purposes, relative expression levels are normalized to time 0. (**g**) Retina total RNA extracted from eyes treated with BSS (control), TGF-β1, 2 or 3 for 21 days. The expression levels of C3, C5 and CD55 were determined by qPCR. The significant difference (*p* < 0.05) is assessed using ANOVA, and paired comparisons between the control, TGF-β1-, TGF-β2-, and TGF-β3-treated groups were conducted using Tukey’s multiple comparison tests. Groups not sharing the same letter are significantly different (*p* < 0.05), as indicated by compact letter display.

Retina pigment epithelial cell (ARPR-19) was treated with TGF-βs at different times; the expression levels of complements 3 and 5 (C3, C5) and decay accelerating factor (DAF, CD55) were determined by qPCR. We found that TGF-βs promoted the expression of C3 (Figure 3a) and C5 (Figure 3b) and inhibited the level of CD55 (Figure 3c). Among all TGF-β isoforms tested, TGF-β2 exhibited the strongest C3- and C5-promoting and CD55-inhibiting activities (Figure 3a–c). To verify that TGF-β2 mediated the upregulation of C3 and C5 and downregulation of CD55, a TGF-β signaling inhibitor, SB431542, was added. SB431542 inhibited the upregulation of C3 (Figure 3d) and C5 (Figure 3e) expression and the downregulation of CD55 (Figure 3f) by TGF-β2. The expression levels of C3, C5, and CD55 in the retina were also validated using qPCR, as shown in Figure 3g. TGF-β2 exhibited the most potent impact on the expression of complement system proteins, surpassing the effects of TGF-β1 and TGF-β3 (Figure 3g). The findings demonstrated that TGF-β2 influences the expression of proteins in the complement system, potentially leading to the advancement of myopia.

### 3.3. Overexpressing CD55 Hinders the Development of Myopia

**Figure 4:**
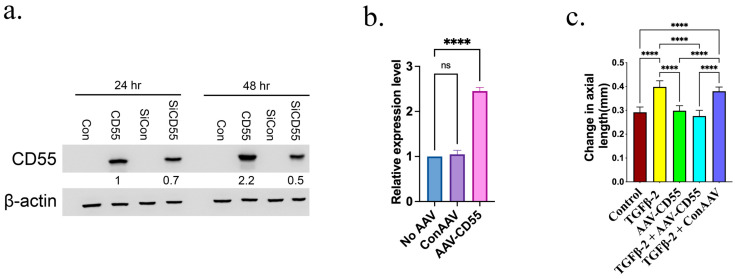
CD55 overexpression inhibited TGF-β2-mediated myopia. (**a**) Assessment of the efficacy of AAV-CD55 (CD55) and siCD55. Viruses ConAAV (control AAV), AAV-CD55 or siCon (siRNA control AAV) infected 293T cells at 1000 multiplicity of infection (MOI) and the cells were harvested at 72 h to determine the expression of CD55. To test the efficacy of siCD55, virus mixtures containing 1000 MOI AAV-CD55 and siCD55 infected 293T cells and the cells were harvested at 72 h to determine the expression of CD55. Western blot analysis was used to determine the level of CD55. Using ImageJ software and the level of β-actin as an internal standard, the relative expression levels were determined. (**b**) Retina total RNA was extracted from eye tissues treated without AAV or with ConAAV and AAV-CD55 (day 7). The expression levels of CD55 were determined by qPCR. (**c**) The change in axial length in eyes treated with BSS (control), TGF-β2, AAV-CD55, TGF-β2 + AAV-CD55 or TGF-β2 + ConCD55. The significance of the difference (*p* < 0.05) is assessed using ANOVA, and paired comparisons were subjected to Tukey’s multiple comparison tests. **** *p* < 0.0001. ns: There is no discernible change.

CD55 is recognized as a suppressor of complement activation. The anaphylatoxins C3a and C5a, produced during the activation of the complement system, could activate NF-κB, which in turn stimulates the expression of NLRP3 and pro-IL-1β, leading to the activation of the inflammasome. We conducted an experiment to determine if increasing the expression of CD55 in the eye could prevent the development of myopia induced by TGF-β2. The AAV-DJ vector was utilized to transport the CD55 transgene into the eye via intravitreal injection. The efficacy of AAV-CD55 in inducing CD55 expression was verified through the infection of 293T cells. Figure 4a demonstrates that AAV-CD55 infection leads to a significant increase in the expression of CD55 protein. We also evaluated the effectiveness of AAV-CD55 in vivo. Retina tissues were obtained 7 days after injecting 1 µL of either control AAV (ConAAV) or AAV-CD55, which had a titer of 1 × 10^12^ GC/mL, into the eye. Retina samples that did not undergo AAV transduction were also collected. CD55 was significantly upregulated in the retina that was injected with AAV-CD55, as compared to the ConAAV or no AAV injection (Figure 4b). The effectiveness of AAV-CD55 in preventing TGF-β2-induced myopia was assessed. The augmentation in axial length caused by TGF-β2 was considerably suppressed by AAV-CD55, as evidenced by the reduced axial length in eyes treated with both TGF-β2 and AAV-CD55, in comparison to eyes treated with TGF-β2 alone or TGF-β2 + ConAAV (Figure 4c). We also found a significant increase in TGF-β and MMP2 and lower collagen 1 expression in the retina, which further confirmed the development of myopia (Supplementary Figure S3).

**Figure 5:**
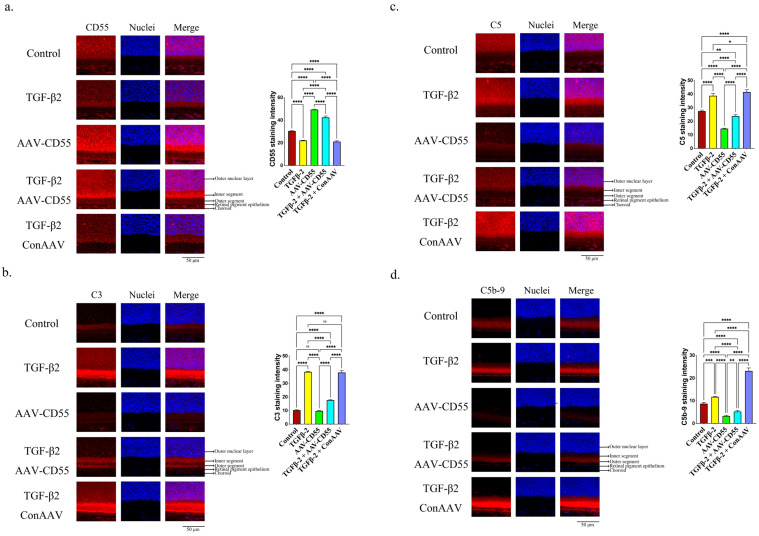
CD55 overexpression inhibited TGF-β2-induced complement activation. Immunofluorescence staining of (**a**) CD55, (**b**) C3, (**c**) C5 and (**d**) C5b-9 in the retina. ImageJ software is employed to ascertain the relative expression levels. The significant difference (*p* < 0.05) is assessed using ANOVA, and paired comparisons between control, TGF-β2-, AAV-CD55-, TGF-β2 + AAV-CD55-, and TGF-β2 + ConAAV-treated eyes in immunofluorescence staining are conducted using Tukey’s multiple comparison tests. * *p* < 0.05, ** *p* < 0.01, *** *p* < 0.001, **** *p* < 0.0001. ns: There is no discernible change.

The administration of AAV-CD55 resulted in an elevation of CD55 (Figure 5a). The overexpression counteracts the promoting effect of TGF-β2 on C3 (Figure 5b) and C5 (Figure 5c). The presence of CD55, C3, and C5 in the retina was verified through qPCR, as shown in Supplementary Figure S4. AAV-CD55 significantly inhibited the increase in deposition of C5b-9 induced by TGF-β2 (Figure 5d).

**Figure 6:**
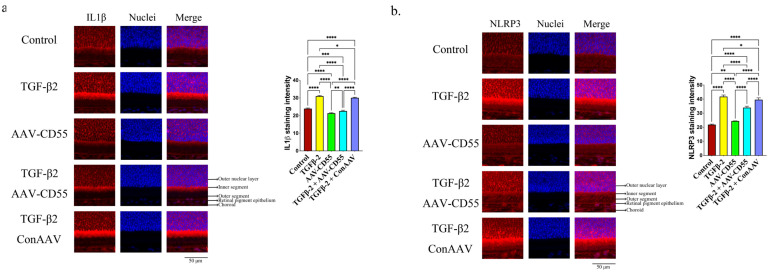
CD55 overexpression inhibited TGF-β2-induced inflammasome activation. Immunofluorescence staining of (**a**) IL-1β and (**b**) NLRP3 in the retina. The ImageJ software is employed to ascertain the relative expression levels. ANOVA is applied to evaluate the significant difference (*p* < 0.05), and Tukey’s multiple comparison tests are used for paired comparisons between control, TGF-β2-, AAV-CD55-, TGF-β2 + AAV-CD55- and TGF-β2 + ConAAV-treated eyes in immunofluorescence staining. * *p* < 0.05, ** *p* < 0.01, *** *p* < 0.001, **** *p* < 0.0001.

Furthermore, the overexpression of CD55 effectively suppressed the TGF-β2-induced production of IL-1β (Figure 6a) and NLRP3 (Figure 6b). The presence of IL-1β and NLRP3 in the retina was also verified through qPCR (Supplementary Figure S4). The findings indicated that upregulation of CD55 could counteract the myopia-enhancing impact caused by TGF-β2.

### 3.4. Inhibiting CD55 Encourages Myopia Development

**Figure 7:**
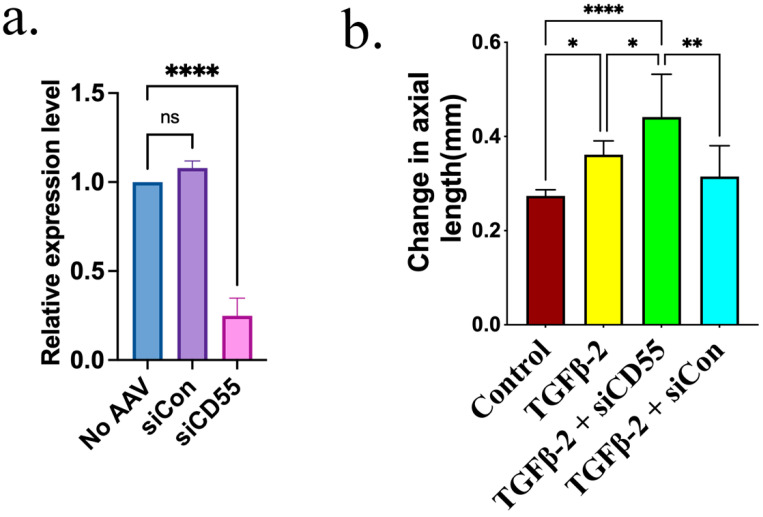
Inhibiting CD55 expression promoted TGF-β2-mediated myopia. (**a**) Retina total RNA was extracted from eye tissues treated without AAV or with siCon and siCD55 (day 7). The expression levels of CD55 were determined by qPCR. (**b**) The change in axial length in eyes treated with BSS (control), TGF-β2, TGF-β2 + siCD55 or TGF-β2 + siCon. ANOVA is applied to evaluate the significant difference (*p* < 0.05), and Tukey’s multiple comparison tests are used for paired comparisons. * *p* < 0.05, ** *p* < 0.01, **** *p* < 0.0001. ns: There is no discernible change.

In order to ascertain the significance of CD55 in the development of myopia caused by TGF-β2, siRNA CD55 was administered using AAV-DJ virus. The effectiveness of AAV-siRNA CD55 (siCD55) in suppressing CD55 expression was confirmed by infecting 293T cells. Figure 4a shows that the infection of siCD55 results in a notable reduction in the expression of CD55 protein induced by AAV-CD55. We assessed the in vivo efficacy of siCD55. Retina tissues were collected 7 days after injecting 1 µL of either siRNA control AAV (siCON) or siCD55, which had a titer of 1 × 10^12^ GC/mL, into the eye. Additionally, samples were collected from the retina that did not undergo AAV transduction. The expression of CD55 was markedly reduced in the retina that received an injection of siCD55, in comparison to the retina that received siCon or no AAV injection (Figure 7a). The efficacy of siCD55 in the induction of myopia by TGF-β2 was evaluated. The increase in axial length induced by TGF-β2 was significantly amplified by siCD55, as demonstrated by the even greater rise in axial length observed in eyes treated with both TGF-β2 and siCD55, compared to eyes treated with TGF-β2 alone or TGF-β2 + siCON (Figure 7b). We also found a significant increase in TGF-β and MMP2 and lower collagen 1 expression in the retina, which further confirmed the development of myopia (Supplementary Figure S5).

**Figure 8:**
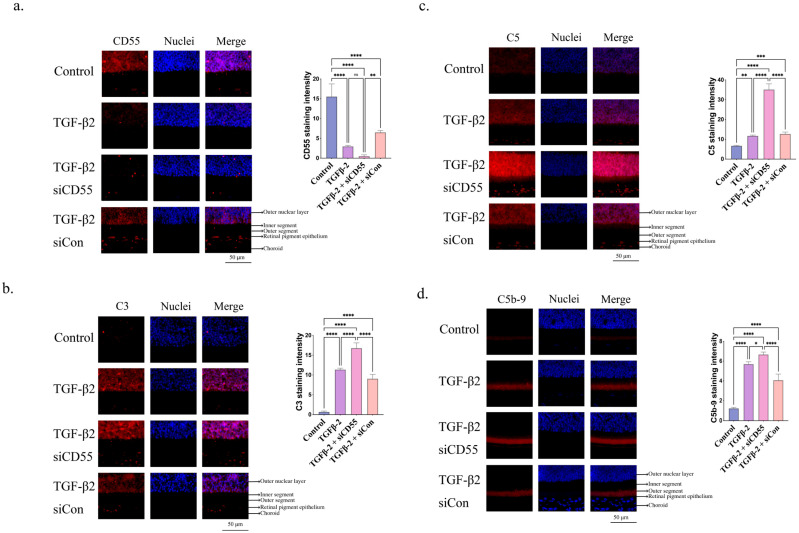
Inhibiting CD55 expression promoted TGF-β2-induced complement activation. Immunofluorescence staining of (**a**) CD55, (**b**) C3, (**c**) C5 and (**d**) C5b-9 in the retina. ImageJ software is employed to ascertain the relative expression levels. ANOVA is applied to evaluate the significant difference (*p* < 0.05), and Tukey’s multiple comparison tests are used for paired comparisons between control, TGF-β2-, TGF-β2 + siCD55- and TGF-β2 + siCon-treated eyes in immunofluorescence staining. * *p* < 0.05, ** *p* < 0.01, *** *p* < 0.001, **** *p* < 0.0001. ns: There is no discernible change.

Administration of siCD55 led to a 4-fold reduction in CD55 levels (Figure 8a). Suppressing the expression of CD55 further intensified the enhancing impact of TGF-β2 on C3 (Figure 8b) and C5 (Figure 8c). The expression of CD55, C3, and C5 in the retina was also evaluated using quantitative polymerase chain reaction (qPCR), as demonstrated in Supplemental Figure S6. siCD55 markedly enhanced the accumulation of C5b-9 triggered by TGF-β2 (Figure 8d).

**Figure 9:**
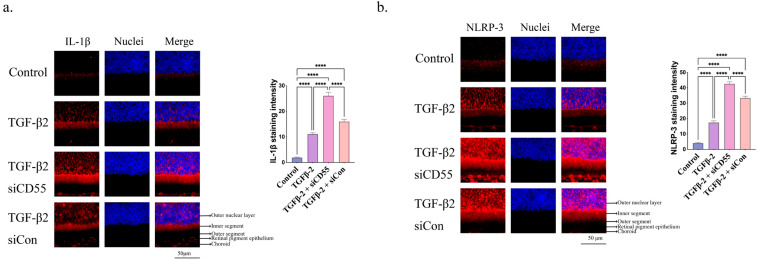
Inhibiting CD55 expression promoted TGF-β2-induced inflammasome activation. Immunofluorescence staining of (**a**) IL-1β and (**b**) NLRP3 in the retina. ImageJ software is employed to ascertain the relative expression levels. ANOVA is applied to evaluate the significant difference (*p* < 0.05), and Tukey’s multiple comparison tests are used for paired comparisons between control, TGF-β2-, TGF-β2 + siCD55- and TGF-β2 + siCon-treated eyes in immunofluorescence staining. **** *p* < 0.0001.

In addition, the suppression of CD55 leads to a notable enhancement in the production of IL-1β (Figure 9a) and NLRP3 (Figure 9b) triggered by TGF-β2. The existence of IL-1β and NLRP3 in the retina was additionally confirmed using qPCR (Supplementary Figure S6). The results indicated the significance of CD55 in the development of myopia induced by TGF-β2.

## 4. Discussion

The study investigates the roles of transforming growth factor-β (TGF-β), the complement system, and inflammasome activation in the pathogenesis of myopia, proposing a significant interplay among these factors that potentially drives disease progression.

The present investigation revealed an elevation in the expression of C3 and C5 caused by TGF-βs, along with the suppression of CD55. Inhibiting TGF-β signaling prevented the elevation of C3 and C5 as well as the down-regulation of CD55. We also found an activation of Smad2 and NF-κB by the treatment of TGF-βs. Increasing NF-κB has been noted in promoting myopia development [28,29,30]. While direct evidence for the TGF-β/Smad2 signaling pathway in promoting C3 and C5 expression is limited, the regulatory capabilities of the TGF-β/Smad pathway suggest a plausible role. TGF-β activates TRAF6 by Smad2, which in turn activates TAK1 (TGF-β-activated kinase 1), leading to the phosphorylation and activation of the IKK (IκB kinase) complex and subsequent NF-κB activation [30]. We found an increase in TAK1 and NF-κB activation in the retina of TGF-β2-treated mice, while the activation was inhibited by CD55 administration (Supplementary Figure S7). TGF-β can activate NF-κB independently of the canonical SMAD pathway. This involves the recruitment and activation of other signaling molecules, such as MAP kinases and PI3K/AKT, which can further activate NF-κB [31]. NF-κB is known to promote the expression of C3 and C5 [25]. The TGF-β/Smad pathway is also known to inhibit expression of certain genes, such as c-Myc [32]. However, how the TGF-β/Smad pathway inhibits CD55 expression is not known. There are three potential mechanisms that the TGF-β/Smad pathway may use to inhibit gene expression. First, Smad2 may bind to the promoters of target genes, directly repressing their transcription. Second, Smad2 may interact with co-repressors and other transcription factors to inhibit gene expression directly. Thirdly, Smad2 may alter chromatin structure, making the promoter regions of certain genes less accessible to the transcriptional machinery. More detailed experiments should be performed to provide molecular mechanisms of either promoting or inhibiting complement protein expression by TGF-β2.

In interpreting the effects of TGF-β2 in our experimental myopia model, it is important to acknowledge that TGF-β is secreted in a latent form and requires extracellular activation to exert its biological functions. This activation process, involving proteolytic cleavage, integrin engagement, or conformational changes in the latent complex, is essential for enabling TGF-β to interact with its receptors. Although we administered recombinant TGF-β2, which is in its active form, we did not directly assess whether latent TGF-β2 requires activation in vivo under physiological or pathological conditions. Importantly, MMP2, which we observed to be upregulated following TGF-β2 treatment, is one of the major proteases capable of activating latent TGF-β. MMP2 cleaves the latency-associated peptide (LAP) from TGF-β, thereby converting it into its bioactive form [33]. This suggests a possible amplification mechanism in our system: the administration of active TGF-β2 may induce MMP2 expression, which in turn promotes further activation of latent TGF-β already present in ocular tissues, especially within the sclera or retinal pigment epithelium. Such positive feedback could sustain or escalate TGF-β signaling, thus contributing to the observed upregulation of complement components and inflammasome activation. This possibility further supports the relevance of MMP2 as both a downstream effector and an upstream enhancer of TGF-β signaling, consistent with its role in scleral remodeling and myopia development. We acknowledge that the dynamics of latent TGF-β activation, particularly in a physiological context, were not fully examined in this study. Future work should include the evaluation of latent versus active TGF-β pools, as well as endogenous activators such as MMP2, integrins (e.g., αvβ6), or thrombospondin-1, to more precisely model the endogenous regulation of TGF-β activity in the progression of myopia.

TGF-β2 is a large protein (~25 kDa); it cannot cross the blood–retina barrier. However, previous studies have demonstrated that subconjunctival injection enables diffusion of proteins and macromolecules into the choroidal circulation via scleral and episcleral vasculature [34]. This provides a plausible route through which TGF-β2 may reach RPE and choroidal targets, consistent with our proposed mechanism. Furthermore, TGF-β is known to possess auto-inductive properties, whereby exogenous TGF-β stimulates endogenous TGF-β expression in a feed-forward loop [35]. This auto-induction could account for the elevated levels of TGF-β2 we observed in the apical compartment following basolateral exposure. We performed a directional secretion assay using ARPE-19 cells cultured on Transwell inserts. This setup mimics the in vivo polarity of RPE cells, forming tight junctions to distinguish apical and basolateral compartments. When active TGF-β2 was applied to the basolateral chamber, we observed a marked increase in TGF-β2 concentration in the apical medium after 24 h. Apically applied TGF-β2 also increased the TGF-β2 concentration in the basolateral medium (Supplementary Figure S8). This directional release supports the physiological relevance of basolateral TGF-β exposure, as would occur via diffusion from the choroidal side. These findings reinforce the concept that subconjunctivally administered TGF-β2, while not directly entering the retina, can stimulate intraocular signaling cascades via the choroid–RPE axis, leading to increased local TGF-β expression and downstream pathological remodeling relevant to myopia progression.

Beyond its role in myopia, TGF-β is involved in a variety of ocular disorders. Its broad impact on cellular processes such as extracellular matrix (ECM) remodeling, differentiation, apoptosis, and proliferation makes it a critical factor in the pathogenesis of several eye conditions. One notable condition where TGF-β is implicated is cataract formation, particularly in high myopia. After cataract surgery, capsular contraction syndrome (CCS) has been linked to greater levels of TGF-β2 in the aqueous humor of myopic eyes. This syndrome is characterized by the shrinkage and opacification of the lens capsule, leading to impaired vision and potential intraocular lens decentration. TGF-β2 promotes the transformation of lens epithelial cells into myofibroblasts, contributing to capsular fibrosis and contraction [36]. In the context of retinal diseases, TGF-β is involved in the progression of proliferative vitreoretinopathy (PVR), a condition that can follow retinal detachment. TGF-β2, in particular, stimulates the proliferation and migration of RPE cells and fibroblasts into the vitreous cavity, leading to the formation of fibrotic membranes that contract and cause recurrent retinal detachments. The signaling pathways activated by TGF-β2 in PVR include the Smad-dependent pathway, which regulates the transcription of genes involved in fibrosis and ECM production [37]. Age-related macular degeneration (AMD) is another condition where TGF-β signaling plays a significant role. In the early stages of AMD, TGF-β helps maintain the homeostasis of the RPE and choroidal tissues. However, in the advanced stages, dysregulated TGF-β signaling can contribute to the formation of choroidal neovascularization, a hallmark of wet AMD. TGF-β promotes angiogenesis through the induction of vascular endothelial growth factor and other pro-angiogenic factors. This neovascularization leads to severe visual impairment if left untreated [38,39]. TGF-β is also involved in the pathogenesis of glaucoma, particularly in the remodeling of the trabecular meshwork (TM) and the optic nerve head. Elevated TGF-β2 levels in the aqueous humor of glaucoma patients induce ECM deposition in the TM, increasing outflow resistance and intraocular pressure. This ECM remodeling is mediated by TGF-β-induced activation of connective tissue growth factor and subsequent fibrotic changes [40]. In summary, TGF-β is a pivotal cytokine in the pathogenesis of various eye diseases, influencing processes from ECM remodeling to cell proliferation and differentiation. Its role in fibrosis and angiogenesis underscores the potential of TGF-β as a therapeutic target. Inhibiting TGF-β signaling pathways could offer new treatment strategies for managing conditions like CCS, PVR, AMD, and glaucoma, thereby preserving vision and improving patient outcomes.

However, the regulatory activity of TGF-β should be noted. TGF-β2 plays a central role in maintaining immune privilege in the eye, primarily through its regulation of regulatory T cells (Tregs) [41]. TGF-β2 is the dominant isoform of TGF-β in the ocular environment, particularly in the aqueous humor, where it contributes to the immunosuppressive milieu necessary to prevent destructive inflammation that could compromise vision. This cytokine is critical in the induction and maintenance of Tregs, which are essential for immune tolerance and preventing autoimmune responses [42]. TGF-β2 induces the expression of Foxp3 in CD4+ T cells, converting them into Tregs capable of suppressing effector T cells and maintaining immune homeostasis [41]. In the eye, RPE cells and other ocular tissues produce TGF-β2, which not only acts locally to suppress immune responses but also promotes the differentiation of Tregs in both the anterior and posterior segments of the eye. These Tregs then suppress bystander T cells through mechanisms that include direct cell-to-cell contact and the secretion of inhibitory cytokines like IL-10 [41]. The protective role of TGF-β2 in ocular immune privilege is further demonstrated in experimental models where the administration of recombinant TGF-β2 can prevent or reduce the severity of autoimmune uveitis by enhancing the population of Tregs in the affected tissues [38]. Therefore, TGF-β2 not only supports immune privilege by directly suppressing inflammation but also by fostering a population of Tregs that perpetuates this immunosuppressive state, highlighting its importance in ocular health and disease.

Accordingly, TGF-β2 functions as positive and negative regulator in the eye. The homeostasis of TGF-β2 is important to maintain normal visual function. Therefore, simply promoting or inhibiting TGF-β2 or its signaling pathway may cause detrimental effects on visual function. It is difficult to determine the most suitable concentration or signaling responses for TGF-β2 for any individual due to the multiple functions of TGF-β2. Blocking the key pathways altered by aberrant TGF-β2 signaling would be a better choice.

The interplay between TGF-β and inflammasome activation, particularly through the NLRP3 pathway, forms a self-perpetuating vicious cycle that significantly contributes to pathological conditions like fibrosis [43]. This process begins with TGF-β enhancing the expression of NLRP3 and pro-IL-1β through the activation of TAK1-NF-κB signaling. Subsequently, TGF-β also increases intracellular ROS levels via the Smad-NOX4 axis, which serves as a secondary signal for the activation of the NLRP3 inflammasome [44]. Once activated, the NLRP3 inflammasome promotes the cleavage of pro-IL-1β into its active form, IL-1β. This IL-1β further stimulates the secretion of TGF-β, creating an autocrine loop that sustains and amplifies both TGF-β production and NLRP3 inflammasome activation [44]. This loop can drive chronic inflammation and fibrosis, as seen in hepatic stellate cells, where the continuous production of TGF-β perpetuates the fibrotic response. Inhibiting key components of this cycle, such as TAK1 or IL-1R, has been shown to disrupt this pathological feedback, highlighting potential therapeutic strategies for conditions driven by this TGF-β–inflammasome axis. We and others have indicated that inflammasome activation promotes myopia development [45,46].

Accordingly, TGF-β2 can activate the inflammasome directly, which does not involve the complement system. CD55 is primarily known for its role in regulating the complement system by preventing the formation of the membrane MAC. However, it has also been implicated in modulating various signaling pathways, including c-Jun N-terminal kinase, Janus kinase/signal transducers and activators of transcription, mitogen-activated protein kinase/NF-κB, and lymphocyte-specific protein tyrosine kinase pathways [47]. Interfering with these pathways, especially the NF-κB signaling pathway, may alter the TGF-β–inflammasome axis.

This study highlights the role of TGF-β2 in promoting myopia through the activation of complement components C3 and C5 and suppression of CD55, leading to enhanced inflammasome activity. The overexpression of CD55 effectively counteracts these effects, suggesting that targeting the complement system, particularly CD55, could be a novel therapeutic strategy for myopia. The findings underscore the complex interplay between TGF-β2, the complement system, and inflammasome activation in myopia development, providing a foundation for future research aimed at developing targeted treatments for this condition.

## Data Availability

All data generated or analyzed during this study are included in this published article and its Supplementary Information files.

## Supplementary Materials

The following supporting information can be downloaded at: https://www.mdpi.com/article/10.3390/cells14161295/s1, Table S1: Primer sequences and universal probe numbers; Figure S1: TGF-β administration promoted the expression of MMP2, while down-regulated collagen I (COL1). The staining intensity was determined using ImageJ software. ANOVA is applied to evaluate the significant difference (*p* < 0.05), and Tukey’s multiple comparison tests are used for paired comparisons between control, TGF-β1, TGF-β2 and TGF-β3-treated groups. Compact letter display was used to displaying the results of multiple pairwise comparisons. Figure S2: TGF-βs promoted inflammasome activation in the eye. Retina total RNA was extracted from eye tissues treated with BSS (control), TGF-β1, TGF-β2 and TGF-β3 (day 21). The expression levels of IL-1β and NLRP3 were determined by qPCR. ANOVA is applied to evaluate the significant difference (*p* < 0.05), and Tukey’s multiple comparison tests are used for paired comparisons between control, TGF-β1, TGF-β2 and TGF-β3-treated eyes in immunofluorescence staining. * *p* < 0.05, **** *p* < 0.0001. Figure S3: CD55 overexpression inhibited TGF-β2 mediated inflammation and myopia. Immunofluorescence staining of (a) TGF-β, (b) MMP2, (c) Collagen1 and (d) TNF-α in the retina. The relative expression levels are determined using the ImageJ software. ANOVA is applied to evaluate the significant difference (*p* < 0.05), and Tukey’s multiple comparison tests are used for paired comparisons between control, TGF-β2, AAV-CD55, TGF-β2 + AAV-CD55 and TGF-β2 + ConAAV-treated eyes in immunofluorescence staining. Compact letter display was used to displaying the results of multiple pairwise comparisons. Figure S4: CD55 overexpression inhibited TGF-β2 induced complement and inflammasome activation. Retina total RNA was extracted from eye tissues. The expression levels of CD55, C3, C5, IL-1β and NLRP3 were determined by qPCR. ANOVA is applied to evaluate the significant difference (*p* < 0.05), and Tukey’s multiple comparison tests are used for paired comparisons between control, T GF-β2, AAV-CD55, TGF-β2 + AAV-CD55 and TGF-β2 + ConAAV-treated eyes. * *p* < 0.05, ** *p* < 0.01, *** *p* < 0.001, **** *p* < 0.0001. Figure S5: Inhibiting CD55 expression promoted TGF-β2 mediated inflammation and myopia. Immunofluorescence staining of (a) TGF-β, (b) MMP2 and (c) TNF-α in the retina. The relative expression levels are determined using the ImageJ software. ANOVA is applied to evaluate the significant difference (*p* < 0.05), and Tukey’s multiple comparison tests are used for paired comparisons between control, TGF-β2, TGF-β2 + siCD55 and TGF-β2 + siCon-treated eyes in immunofluorescence staining. * *p* < 0.05, ** *p* < 0.01, *** *p* < 0.001, **** *p* < 0.0001. Figure S6: Inhibiting CD55 expression promoted TGF-β2 induced complement and inflammasome activation. Retina total RNA was extracted from eye tissues. The expression levels of CD55, C3, C5, IL-1β and NLRP3 were determined by qPCR. ANOVA is applied to evaluate the significant difference (*p* < 0.05), and Tukey’s multiple comparison tests are used for paired comparisons between control, TGF-β2, TGF-β2 + siCD55 and TGF-β2 + siCon-treated eyes. * *p* < 0.05, ** *p* < 0.01, *** *p* < 0.001, **** *p* < 0.0001. Figure S7: CD55 modulates the TGF-β2 mediated TAK1 and NF-κB activation. Retina tissues were collected on day 21. Western blot analysis was used to determine the levels of phospho-NFκB (Ser 536), NFκB, phospho-TAK1 (Thr187). The relative expression levels were determined using ImageJ software and using the level of β-actin as internal standard. Figure S8: Directional secretion of TGF-β2 across polarized ARPE-19 cells. Polarized ARPE-19 cells were cultured on transwell inserts in 12-well plates to establish tight junctions. Cells were treated with recombinant TGF-β2 (at indicated concentrations) applied to either the apical or basolateral chamber for 24 h. The concentration of TGF-β2 was then quantified in the opposite chamber using ELISA to assess trans-epithelial transport or secretion. The labels “Basolateral” and “Apical” in the figure indicate the side where TGF-β2 was originally added, with measurements taken from the opposite side. Statistical analysis was performed using one-way ANOVA followed by Tukey’s multiple comparison test. Groups not sharing the same letter are significantly different (*p* < 0.05), as indicated by compact letter display.

## Abbreviations

The following abbreviations are used in this manuscript:

TGF-β2	Transforming Growth Factor (TGF)-β2
AAV	Adeno-Associated Virus
qPCR	Real-Time Quantitative PCR
NF-κB	Nuclear Factor κB
IL-1β	Interleukin 1β
NLRP3	NLR Family Pyrin Domain Containing 3
MMP2	Matrix Metalloproteinase 2
GAPDH	Glyceraldehyde 3-phosphate Dehydrogenase
RPIA	Radioimmunoprecipitation
ECM	Extracellular Matrix
CCS	Capsular Contraction Syndrome
PVR	Proliferative Vitreoretinopathy
AMD	Age-related Macular Degeneration
TM	Trabecular Meshwork
TAK1	TGF-β-activated kinase 1

## References

[1] Morgan I.G., Ohno-Matsui K., Saw S.M. (2012). Myopia. Lancet.

[2] Resnikoff S., Pascolini D., Mariotti S.P., Pokharel G.P. (2008). Global Magnitude of Visual Impairment Caused by Uncorrected Refractive Errors in 2004. Bull. World Health Organ..

[3] McCarty C.A. (2006). Uncorrected Refractive Error. Br. J. Ophthalmol..

[4] Leo S.W., Young T.L. (2011). An Evidence-Based Update on Myopia and Interventions to Retard Its Progression. J. AAPOS.

[5] Kaiti R., Shyangbo R., Sharma I.P., Dahal M. (2021). Review on Current Concepts of Myopia and Its Control Strategies. Int. J. Ophthalmol..

[6] World Health Organization (2000). Global Initiative for the Elimination of Avoidable Blindness.

[7] Wang M.K., Sun H.Q., Xiang Y.C., Jiang F., Su Y.P., Zou Z.M. (2012). Different Roles of TGF-Beta in the Multi-Lineage Differentiation of Stem Cells. World J. Stem Cells.

[8] Asano K., Shikama Y., Shoji N., Hirano K., Suzaki H., Nakajima H. (2010). Tiotropium Bromide Inhibits TGF-β-Induced MMP Production from Lung Fibroblasts by Interfering with Smad and MAPK Pathways in Vitro. Int. J. Chron. Obs. Pulm. Dis..

[9] Jobling A.I., Nguyen M., Gentle A., McBrien N.A. (2004). Isoform-Specific Changes in Scleral Transforming Growth Factor-Beta Expression and the Regulation of Collagen Synthesis During Myopia Progression. J. Biol. Chem..

[10] Chen B.-Y., Wang C.-Y., Chen W.-Y., Ma J.-X. (2013). Altered TGF-Β2 and bFGF Expression in Scleral Desmocytes from an Experimentally-Induced Myopia Guinea Pig Model. Graefes Arch. Clin. Exp. Ophthalmol..

[11] Zhuang H., Zhang R., Shu Q., Jiang R., Chang Q., Huang X., Jiang C., Xu G. (2014). Changes of TGF-Β2, MMP-2, and TIMP-2 Levels in the Vitreous of Patients with High Myopia. Graefes Arch. Clin. Exp. Ophthalmol..

[12] He L., Frost M.R., Siegwart J.T., Norton T.T. (2014). Gene Expression Signatures in Tree Shrew Choroid in Response to Three Myopiagenic Conditions. Vis. Res..

[13] Ku H., Chen J.J.-Y., Chen W., Tien P.-T., Lin H.-J., Wan L., Xu G. (2024). The Role of Transforming Growth Factor Beta in Myopia Development. Mol. Immunol..

[14] Wang Y., Tang Z., Xue R., Singh G.K., Lv Y., Shi K., Cai K., Deng L., Yang L. (2011). TGF-Β1 Promoted MMP-2 Mediated Wound Healing of Anterior Cruciate Ligament Fibroblasts Through NF-κB. Connect. Tissue Res..

[15] Clark S.J., Bishop P.N. (2017). The Eye as a Complement Dysregulation Hotspot. Semin. Immunopathol..

[16] Long Q., Ye J., Li Y., Wang S., Jiang Y. (2013). C-Reactive Protein and Complement Components in Patients with Pathological Myopia. Optom. Vis. Sci..

[17] Gao T.T., Long Q., Yang X. (2015). Complement Factors C1q, C3 and C5b-9 in the Posterior Sclera of Guinea Pigs with Negative Lens-Defocused Myopia. Int. J. Ophthalmol..

[18] Riddell N., Crewther S.G. (2017). Novel Evidence for Complement System Activation in Chick Myopia and Hyperopia Models: A Meta-Analysis of Transcriptome Datasets. Sci. Rep..

[19] Heeger P.S., Lalli P.N., Lin F., Valujskikh A., Liu J.B., Muqim N., Xu Y.Y., Medof M.E. (2005). Decay-Accelerating Factor Modulates Induction of T Cell Immunity. J. Exp. Med..

[20] Esposito A., Suedekum B., Liu J., An F., Lass J., Strainic M.G., Lin F., Heeger P., Medof M.E. (2010). Decay Accelerating Factor Is Essential for Successful Corneal Engraftment. Am. J. Transpl..

[21] Cocuzzi E., Szczotka L.B., Brodbeck W.G., Bardenstein D.S., Wei T., Medof M.E. (2001). Tears Contain the Complement Regulator CD59 as Well as Decay-Accelerating Factor (DAF). Clin. Exp. Immunol..

[22] Xu S., Wang D., Tan L., Lu J. (2024). The Role of NLRP3 Inflammasome in Type 2 Inflammation Related Diseases. Autoimmunity.

[23] Niyonzima N., Halvorsen B., Sporsheim B., Garred P., Aukrust P., Mollnes T.E., Espevik T. (2017). Complement Activation by Cholesterol Crystals Triggers a Subsequent Cytokine Response. Mol. Immunol..

[24] Arbore G., Kemper C. (2016). A Novel “Complement–Metabolism–Inflammasome Axis” as a Key Regulator of Immune Cell Effector Function. Eur. J. Immunol..

[25] Liu M., Wang H., Zhang J., Yang X., Li B., Wu C., Zhu Q. (2018). NF-κB Signaling Pathway-Enhanced Complement Activation Mediates Renal Injury in Trichloroethylene-Sensitized Mice. J. Immunotoxicol..

[26] Kaneko N., Kurata M., Yamamoto T., Morikawa S., Masumoto J. (2019). The Role of Interleukin-1 in General Pathology. Inflamm. Regen..

[27] Markert E.K., Klein H., Viollet C., Rust W., Strobel B., Kauschke S.G., Makovoz B., Neubauer H., Bakker R.A., Blenkinsop T.A. (2022). Transcriptional Comparison of Adult Human Primary Retinal Pigment Epithelium, Human Pluripotent Stem Cell-Derived Retinal Pigment Epithelium, and ARPE19 Cells. Front. Cell Dev. Biol..

[28] Wei C.-C., Lin H.-J., Lim Y.-P., Chen C.-S., Chang C.-Y., Lin C.-J., Chen J.J.-Y., Tien P.-T., Lin C.-L., Wan L. (2019). PM2.5 and NOx Exposure Promote Myopia: Clinical Evidence and Experimental Proof. Environ. Pollut..

[29] Wei C.-C., Kung Y.-J., Chen C.S., Chang C.-Y., Lin C.-J., Tien P.-T., Chang H.-Y., Chen H.-J., Huang Y.-S., Lin H.-J. (2018). Allergic Conjunctivitis-Induced Retinal Inflammation Promotes Myopia Progression. EBioMedicine.

[30] Lin H.-J., Wei C.-C., Chang C.-Y., Chen T.-H., Hsu Y.-A., Hsieh Y.-C., Chen H.-J., Wan L. (2016). Role of Chronic Inflammation in Myopia Progression: Clinical Evidence and Experimental Validation. EBioMedicine.

[31] Deng Z., Fan T., Xiao C., Tian H., Zheng Y., Li C., He J. (2024). TGF-β Signaling in Health, Disease, and Therapeutics. Signal Transduct. Target. Ther..

[32] Yagi K., Furuhashi M., Aoki H., Goto D., Kuwano H., Sugamura K., Miyazono K., Kato M. (2002). C-Myc Is a Downstream Target of the Smad Pathway. J. Biol. Chem..

[33] Yu Q., Stamenkovic I. (2000). Cell Surface-Localized Matrix Metalloproteinase-9 Proteolytically Activates TGF-β and Promotes Tumor Invasion and Angiogenesis. Genes. Dev..

[34] Raghava S., Hammond M., Kompella U.B. (2004). Periocular Routes for Retinal Drug Delivery. Expert. Opin. Drug Deliv..

[35] Hariyanto N.I., Yo E.C., Wanandi S.I. (2021). Regulation and Signaling of TGF-β Autoinduction. Int. J. Mol. Cell. Med..

[36] Zhang K., Zhu X., Chen M., Sun X., Yang J., Zhou P., Lu Y. (2016). Elevated Transforming Growth Factor-Β2 in the Aqueous Humor: A Possible Explanation for High Rate of Capsular Contraction Syndrome in High Myopia. J. Ophthalmol..

[37] Callan A., Jha S., Valdez L., Baldado L., Tsin A. (2024). TGF-β Signaling Pathways in the Development of Diabetic Retinopathy. Int. J. Mol. Sci..

[38] Liu D., Zhang C., Zhang J., Xu G.-T., Zhang J. (2023). Molecular Pathogenesis of Subretinal Fibrosis in Neovascular AMD Focusing on Epithelial-Mesenchymal Transformation of Retinal Pigment Epithelium. Neurobiol. Dis..

[39] Higashijima F., Hasegawa M., Yoshimoto T., Kobayashi Y., Wakuta M., Kimura K. (2022). Molecular Mechanisms of TGFβ-Mediated EMT of Retinal Pigment Epithelium in Subretinal Fibrosis of Age-Related Macular Degeneration. Front. Ophthalmol..

[40] Hernandez H., Roberts A.L., McDowell C.M. (2020). Nuclear Factor-Kappa Beta Signaling Is Required for Transforming Growth Factor Beta-2 Induced Ocular Hypertension. Exp. Eye Res..

[41] Keino H., Horie S., Sugita S. (2018). Immune Privilege and Eye-Derived T-Regulatory Cells. J. Immunol. Res..

[42] Matta B., Bora P.S., Neuhouser A.J., Bora N.S. (2019). Inhibitory Role of Transforming Growth Factor Β2 in Experimental Autoimmune Anterior Uveitis. Graefes Arch. Clin. Exp. Ophthalmol..

[43] Alyaseer A.A.A., de Lima M.H.S., Braga T.T. (2020). The Role of NLRP3 Inflammasome Activation in the Epithelial to Mesenchymal Transition Process During the Fibrosis. Front. Immunol..

[44] Kang H., Seo E., Oh Y.S., Jun H.-S. (2022). TGF-β Activates NLRP3 Inflammasome by an Autocrine Production of TGF-β in LX-2 Human Hepatic Stellate Cells. Mol. Cell. Biochem..

[45] Chen C.S., Lin C.-F., Lee D.-Y., Tien P.-T., Wang Y.-C., Chang C.Y., Lin E.-S., Wu M.Y., Ku H., Gan D. (2023). Acupuncture Modulates Development of Myopia by Reducing NLRP3 Inflammasome Activation via the Dopamine-D1R Signaling Pathway. Acupunct. Med..

[46] Xiao K., Chen Z., He S., Long Q. (2024). Up-Regulation of Scleral C5b-9 and Its Regulation of the NLRP3 Inflammasome in a Form-Deprivation Myopia Mouse Model. Immunobiology.

[47] Bharti R., Dey G., Lin F., Lathia J., Reizes O. (2022). CD55 in Cancer: Complementing Functions in a Non-Canonical Manner. Cancer Lett..

