# Supplementary material for: The Function of Transforming Growth Factor 2 in Facilitating Inflammasome Activation to Enhance the Development of Myopia via Complement System
Source: Cells. 2025 Aug 20;14(16):1295. doi: 10.3390/cells14161295 (PMC12385021; doi:10.3390/cells14161295)

Supplementary Table S1: Primer sequences and universal probe numbers

**Gene**	Accession	Left primer	Right primer	Probe no.
**Rat**				
**C3**	NM_016994	CGCACCAAGAAGGAGGGTAT	CATAGTGCTGTAGGGCTGGG	49
**C5**	NM_053020	CGCGGCTCCTGGTCTATTAC	TTATCCAGACTGCGTCAGCC	32
**CD55**	NM_022269	CTGTCCCCGACTGTAAGCG	TGTTTACCCGAGTTTGGCGT	10
**IL-1β**	NM_031512	TGACTTCACCATGGAACCCG	CATGTCCTGGGGAAGGCATT	46
**Nlrp3**	XM_006246453	TCTGCTGGATTGTGTGCACA	GTCGTGGTCTTGGAGGTCTG	84
**Human**				
**C3**	NM_000064	TACTACACGCTGATCGGTGC	CAGGAGTCCTTGACGTCCAC	49
**C5**	NM_001317163	ACGCATAGTAGCATGTGCCA	TGTCCATCACCGCATGAGAG	3
**CD55**	NM_000574	ATGAAGGAGAGTGGAGTGGC	TGTTGGTGGGACCTTGGAAG	6
**IL-1β**	NM_000576	TCCGGGACTCACAGCAAAAA	TATCCTGTCCCTGGAGGTGG	93
**Nlrp3**	NM_001079821	TTGACGAGCACATAGGACCG	CTGATGAGGCTGCTCAGGAG	29

C3: Complement 3; C5: Complement 5; IL-1β: Interleukin 1β; Nlrp3: NLR family, pyrin domain containing 3

Supplementary Figure S1: TGF-β administration promoted the expression of MMP2, while down-regulated collagen I (COL1). The staining intensity was determined using Image J software. ANOVA is applied to evaluate the significant difference ($P < 0.05$), and Tukey's multiple comparison tests are used for paired comparisons between control, TGF-β1, TGF-β2 and TGF-β3-treated groups. Compact letter display was used to displaying the results of multiple pairwise comparisons.

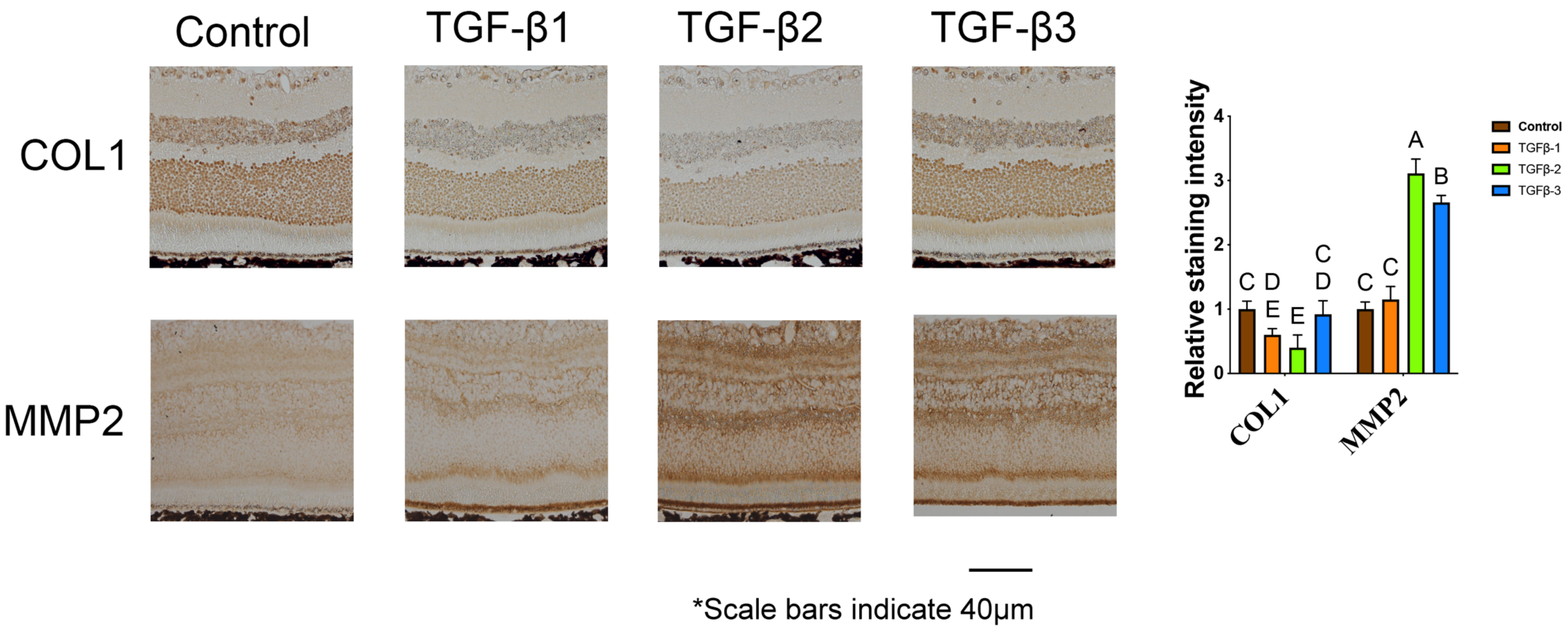

Supplementary Figure S2: TGF-βs promoted inflammasome activation in the eye. Retina total RNA was extracted from eye tissues treated with BSS (control), TGF-β1, TGF-β2 and TGF-β3 (day 21). The expression levels of IL-1β and NLRP3 were determined by qPCR. ANOVA is applied to evaluate the significant difference ($P < 0.05$), and Tukey's multiple comparison tests are used for paired comparisons between control, TGF-β1, TGF-β2 and TGF-β3-treated eyes in immunofluorescence staining. * $P < 0.05$, **** $P < 0.0001$.

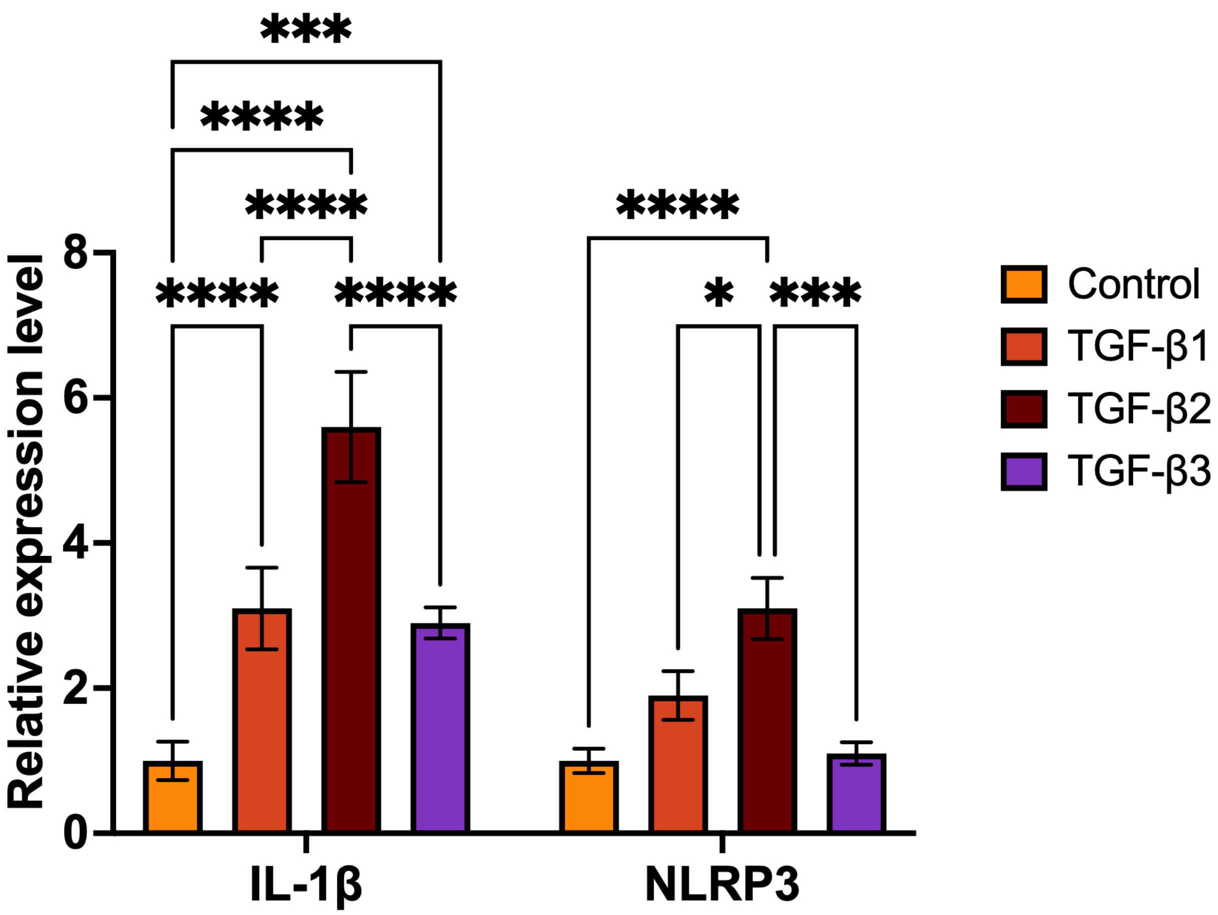

Supplementary Figure S3: CD55 overexpression inhibited TGF-β2 mediated inflammation and myopia. Immunofluorescence staining of (a)TGF-β, (b) MMP2, (c) Collagen1 and (d) TNF-α in the retina. The relative expression levels are determined using the Image J software. ANOVA is applied to evaluate the significant difference ($P < 0.05$), and Tukey's multiple comparison tests are used for paired comparisons between control, TGF-β2, AAV-CD55, TGF-β2 + AAV-CD55 and TGF-β2 + ConAAV-treated eyes in immunofluorescence staining. Compact letter display was used to displaying the results of multiple pairwise comparisons.

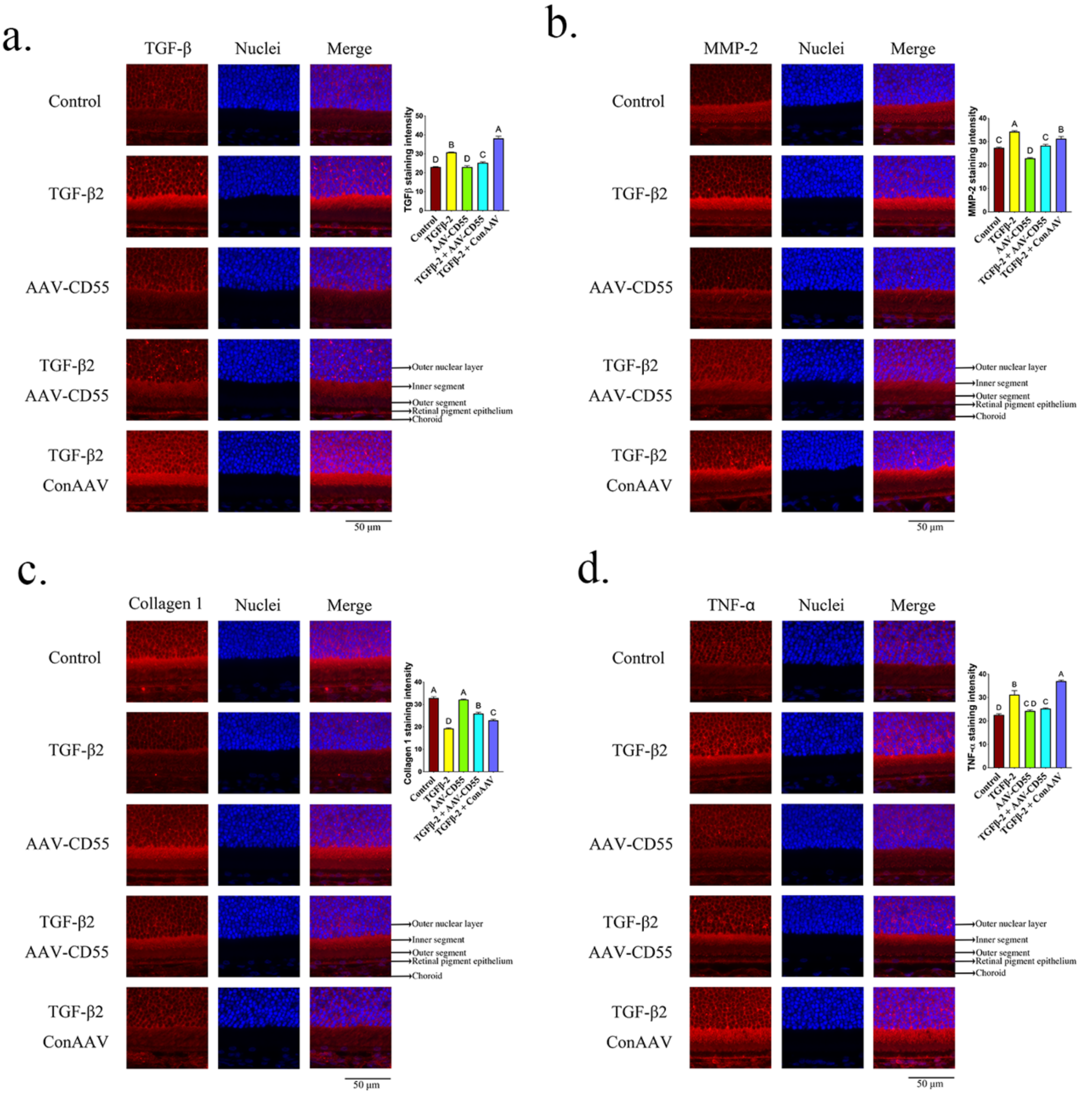

Supplementary Figure S4: CD55 overexpression inhibited TGF-β2 induced complement and inflammasome activation. Retina total RNA was extracted from eye tissues. The expression levels of CD55, C3, C5, IL-1β and NLRP3 were determined by qPCR. ANOVA is applied to evaluate the significant difference ($P < 0.05$), and Tukey's multiple comparison tests are used for paired comparisons between control, TGF-β2, AAV-CD55, TGF-β2 + AAV-CD55 and TGF-β2 + ConAAV-treated eyes. * $P < 0.05$, ** $P < 0.01$, *** $P < 0.001$, **** $P < 0.0001$.

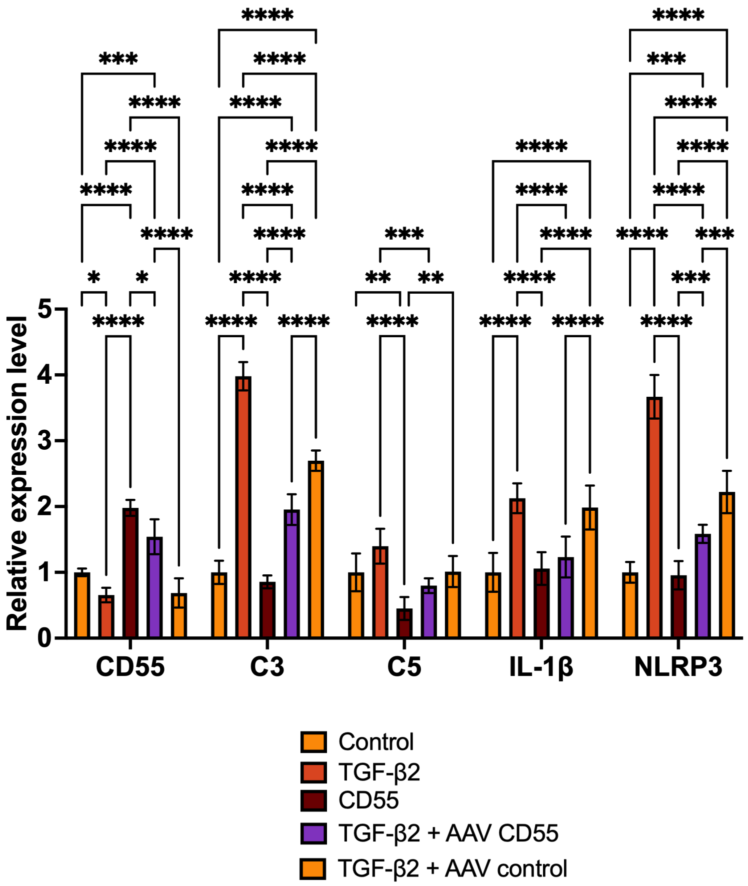

Supplementary Figure S5: Inhibiting CD55 expression promoted TGF-β2 mediated inflammation and myopia. Immunofluorescence staining of (a)TGF-β, (b) MMP2 and (c) TNF-α in the retina. The relative expression levels are determined using the Image J software. ANOVA is applied to evaluate the significant difference ($P < 0.05$), and Tukey's multiple comparison tests are used for paired comparisons between control, TGF-β2, TGF-β2 + siCD55 and TGF-β2 + siCon-treated eyes in immunofluorescence staining. * $P < 0.05$, ** $P < 0.01$, *** $P < 0.001$, **** $P < 0.0001$.

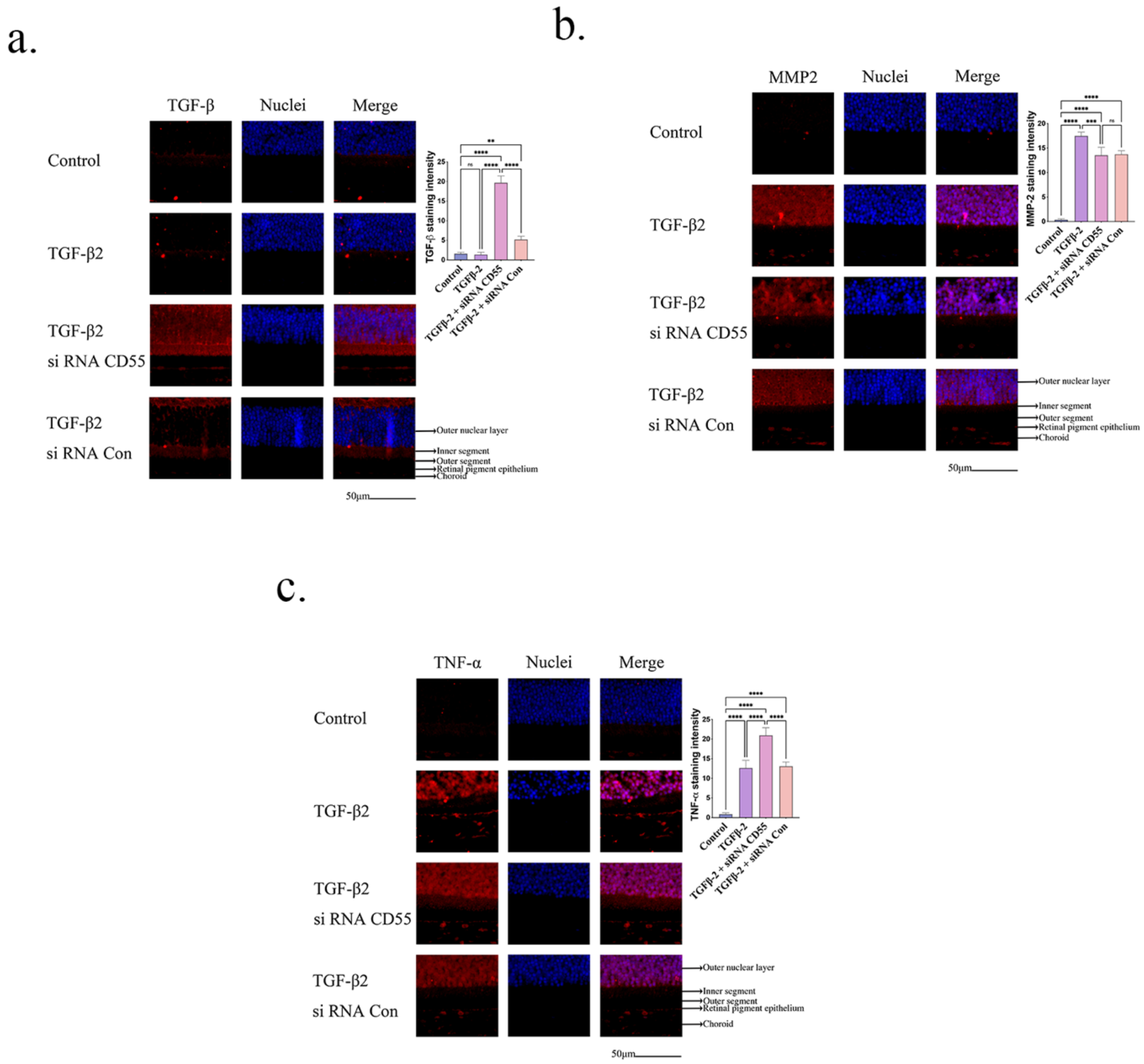

Supplementary Figure S6: Inhibiting CD55 expression promoted TGF-β2 induced complement and inflammasome activation. Retina total RNA was extracted from eye tissues. The expression levels of CD55, C3, C5, IL-1β and NLRP3 were determined by qPCR. ANOVA is applied to evaluate the significant difference ($P < 0.05$), and Tukey's multiple comparison tests are used for paired comparisons between control, TGF-β2, TGF-β2 + siCD55 and TGF-β2 + siCon-treated eyes. * $P < 0.05$, ** $P < 0.01$, *** $P < 0.001$, **** $P < 0.0001$.

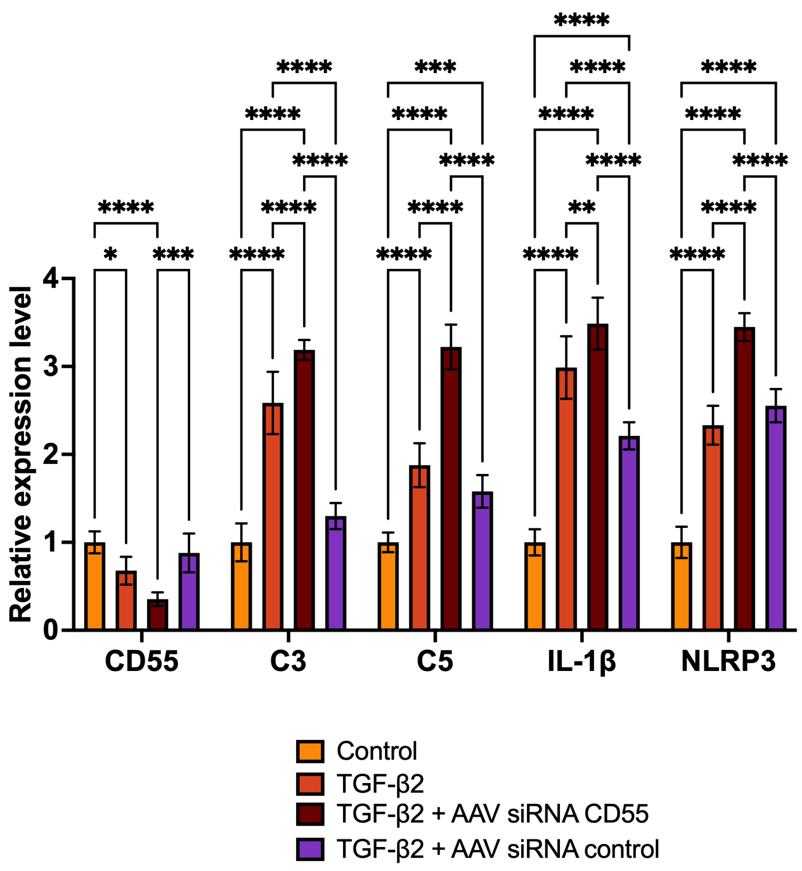

Supplementary Figure S7: CD55 modulates the TGF-β2 mediated TAK1 and NF-κB activation. Retina tissues were collected on day 21. Western blot analysis was used to determine the levels of phospho-NFκB (Ser 536), NFκB, phospho-TAK1 (Thr187). The relative expression levels were determined using ImageJ software and using the level of β-actin as internal standard.

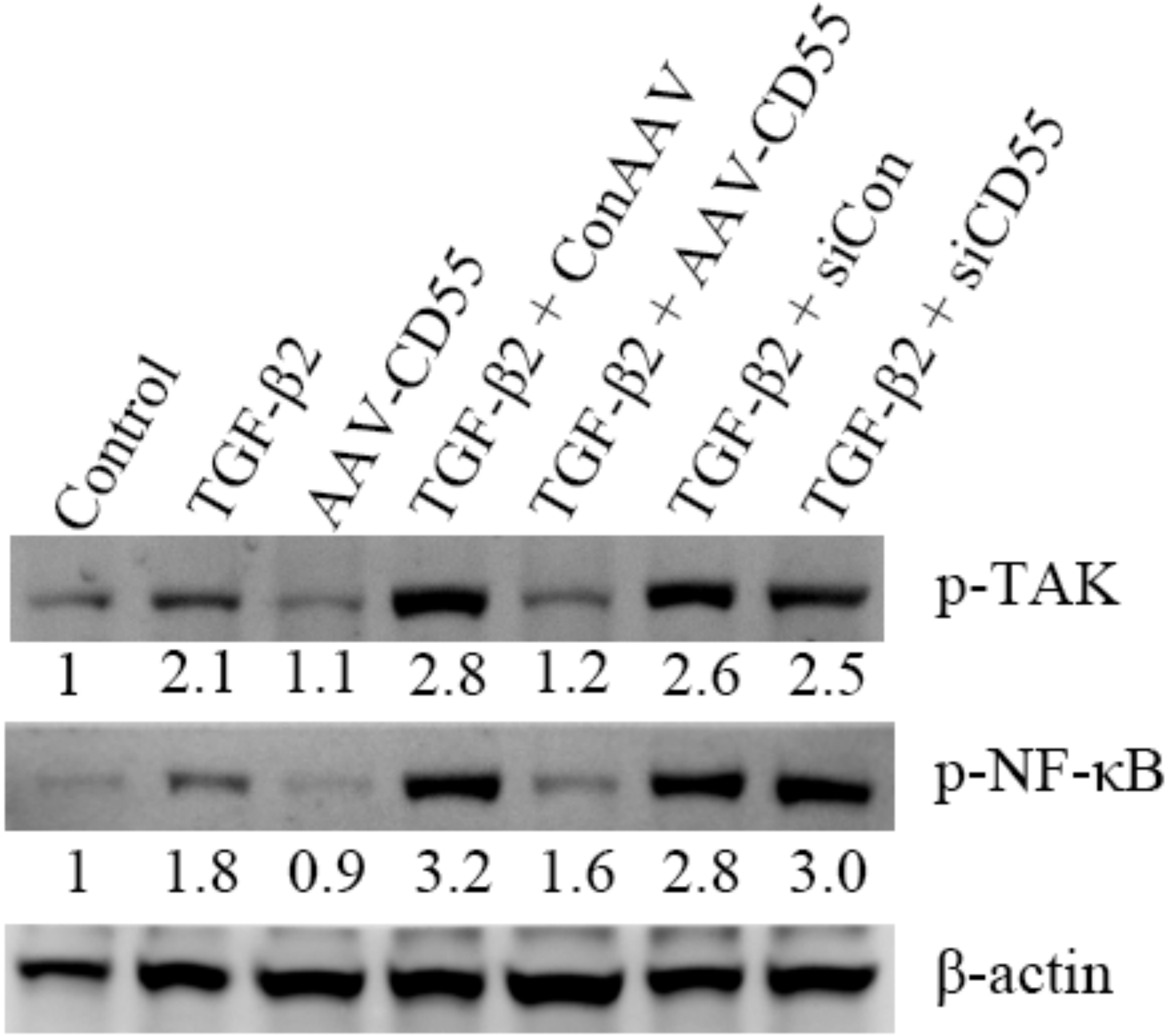

Supplementary Figure S8: Directional secretion of TGF-β2 across polarized ARPE-19 cells. Polarized ARPE-19 cells were cultured on transwell inserts in 12-well plates to establish tight junctions. Cells were treated with recombinant TGF-β2 (at indicated concentrations) applied to either the apical or basolateral chamber for 24 hours. The concentration of TGF-β2 was then quantified in the opposite chamber using ELISA to assess trans-epithelial transport or secretion. The labels "Basolateral" and "Apical" in the figure indicate the side where TGF-β2 was originally added, with measurements taken from the opposite side. Statistical analysis was performed using one-way ANOVA followed by Tukey's multiple comparison test. Groups not sharing the same letter are significantly different ($P < 0.05$), as indicated by compact letter display.

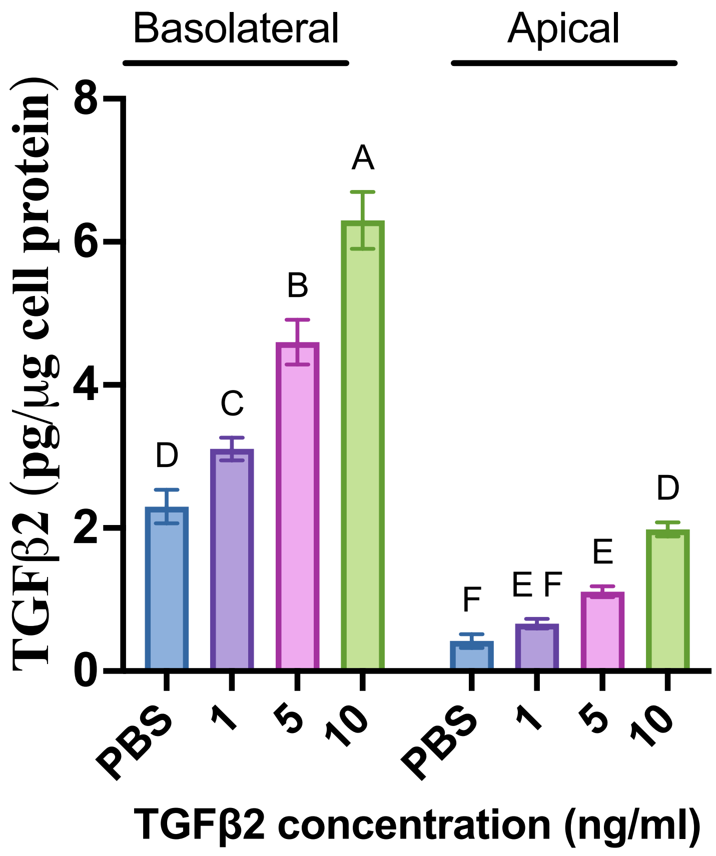

Supplement: Supplementary file 1 [file cells-14-01295-s001.zip › cells-3674958-supplementary.pdf]
